# *Env* diversity-dependent protection of the attenuated equine infectious anaemia virus vaccine

**DOI:** 10.1080/22221751.2020.1773323

**Published:** 2020-06-11

**Authors:** Yuezhi Lin, Xue-Feng Wang, Yuhong Wang, Cheng Du, Huiling Ren, Cong Liu, Dantong Zhu, Jie Chen, Lei Na, Diqiu Liu, Zhibiao Yang, Xiaojun Wang

**Affiliations:** a State Key Laboratory of Veterinary Biotechnology, Harbin Veterinary Research Institute of Chinese Academy of Agricultural Sciences, Harbin, People’s Republic of China; b Shanghai Key Laboratory of Veterinary Biotechnology, Shanghai, People’s Republic of China; c Department of Geriatrics and Gerontology, First Affiliated Hospital of Harbin Medical University, Harbin, People’s Republic of China

**Keywords:** EIAV, *env*, diversity, immune protection, vaccine, retrovirus

## Abstract

Lentiviruses harbour high genetic variability for efficient evasion from host immunity. An attenuated equine infectious anaemia (EIA) vaccine was developed decades ago in China and presented remarkably robust protection against EIA. The vaccine was recently proven to have high genomic diversity, particular in *env*. However, how and to what extent the high *env* diversity relates to immune protection remains unclear. In this study, we compared immune protections and responses of three groups of horses stimulated by the high-diversity vaccine EIAV_HD, a single molecular clone of the vaccine EIAV_LD with low *env* diversity, as well as a constructed vaccine strain EIAV_MD with moderate *env* diversity. The disparity of virus-host interactions between three *env* diversity-varied groups (5 horses in each group) was evaluated using clinical manifestation, pathological scores, and *env*-specific antibody. We found the highest titres of *env* antibodies (Abs) or neutralizing Abs (nAbs) in the EIAV_HD group, followed by the EIAV_MD group, and the lowest titres in the EIAV_LD group (*P*<0.05). The occurrence of disease/death was different between EIAV_HD group (1/0), EIAV_MD (2/2), and EIAV_LD group (4/2). A similar *env* diversity-related linear relationship was observed in the clinical manifestations and pathological changes. This diversity-dependent disparity in changes between the three groups was more distinct after immunosuppression, suggesting that *env* diversity plays an important role in protection under low host immunocompetence. In summary, inoculation with vaccines with higher genetic diversity could present broader and more efficient protection. Our findings strongly suggest that an abundance of Env antigens are required for efficient protection against lentiviruses.

## Introduction

An attenuated vaccine against the non-primate lentivirus equine infectious anaemia (EIAV) has been used to provide efficacious protection against natural EIAV infection for decades in China [1,2]. This vaccine was developed from a natural virulent strain of EIAV by gradually attenuating it in donkey monocytes via 121 passages *in vitro*, and was thereafter called EIAV_DLV121_ [3,4]. The substantial potency of the EIAV_DLV121_ vaccine could be demonstrated by the protection of animals against the challenge by a supervirulent EIAV strain (EIAV_LN40_), which otherwise resulted in approximately 100% mortality [1,2,4]. The attenuated EIAV vaccine (EIAV_DLV121_) could provide an intriguing cross-variant protection, with 85% protection against EIAV_LN40_ or North American EIAV, which has never been observed in vaccines against other lentiviral diseases such as HIV-1 or SIV [5–7].

*Env* is the most diverse gene in the lentiviral genome. It has been reported that when the viral *env* gene diverges 13% from that of the vaccine strain, the protective ratio of EIAV_D9_ (an attenuated vaccine) was reduced from 100% to 0% compared to the homogenous strain [8]. The North American and the Chinese EIAV strains show a 32% *env* heterogeneity [2,3,9]. Systematic sequencing analysis showed that the genomic diversity of the attenuated vaccine EIAV_DLV121_ was 2.5 times higher than that of its parental strain (2.07% vs 0.81%). Our previous studies suggested that the vaccine EIAV_DLV121_ might be evolved from a natural quasispecies [2,3,10]. Such intriguingly high diversity in the EIAV_DLV121_ vaccine was perhaps acquired through an evolutionary process during the long-time passaging. In the genome of the EIAV_DLV121_ strain, the highest diversity is seen in the *env* gene, which displays 4 times higher diversity than that of its parent strain (2.4% vs 0.6%) [10]. It has been well documented that the considerably variable *env* plays a dominant role in virus-to-host immunity [8,11–13], and it has become a target in the development of efficacious lentivirus vaccines [8,13–16]. Therefore, the potent attenuated lentivirus vaccine harbouring high *env* diversity was potentially an ideal candidate in response to the ongoing variation of EIAV [1,8,11]. Although there has been some speculation that the efficacy of the EIAV vaccine was related to the diversity of this attenuated strain, especially for *env*, there is, to date, no direct data that clearly supports this hypothesis.

We therefore aimed to identify how and to what extent the high diversity of *env* in the vaccine EIAV_DLV121_ plays a role during protection. This study was designed to investigate the potential correlations between the *env* diversity (generated through long-term passaging) and protection against EIA. The high-diversity vaccine EIAV_DLV121_ (hereafter termed EIAV_HD), a single molecular clone of vaccine with low genome diversity (hereafter termed EIAV_LD), and a constructed *env* moderate-diversity vaccine strain (termed EIAV_MD) were used to vaccinate three groups of horses. We assessed the virus-host interactions over a long timescale. Our results show that the protection rate against fatal challenging, the clinical manifestation, pathological scores, and *env*-specific antibody, are all positively correlated with *env* diversity in the vaccine strain, indicating that higher genetic diversity of vaccines could present broader and more efficient protection.

## Materials and methods

### Ethics

The horses used in the inoculation-and-challenge study were approved by the Harbin Veterinary Research Institute (HVRI), the Chinese Academy of Agricultural Sciences (CAAS). The Animal Ethics Committee approval number is Heilongjiang-SYXK (Hei) 2017-009. The horses used in the immunization studies were treated strictly in accordance with the Principles of Laboratory Animals of the Ministry of Science and Technology of China. All physical procedures associated with this work were done under anaesthesia to minimize pain and distress in accordance with the recommendations of the Ethics Committees of HVRI.

### Construction and verification of a platform involving *env* diversity-variant stains *in vitro*

To construct *env* diversity-varied EIAV strains *in vitro*, the attenuated vaccine strain of EIAV_DLV121_ was used as the strain with high diversity (termed EIAV_HD). An infectious clone, which had been previously constructed using a single EIAV genome, was used as a control strain representing a low-diversity EIAV strain (termed EIAV_LD). The EIAV vaccine strain and the infectious clone were prepared from equine macrophage cells (eMDM) as described previously [17]. Using these two strains as templates, a “hybrid” strain was created using intracellular homologous recombination [18]. Briefly, the backbone *gag-pol* gene derived from the infectious clone was obtained by PCR (CMV-F: 5′-TAGTTATTAATAGTAATCAATTACGGGGTCATTAGT-3′ and RRE-R: 5′-GTTAGTTAGTAAATGACCTACACCCAGGAAATGAACCCCA-3′). The complete *env* gene and the 3′ LTR region were derived from the vaccine using the primer pair 5′-CCACCAGAGTGTTGTGGAAAGGTGA-3′ and 5′-TGTTAGATCTTGAAAACAAGAC-3′. The two PCR products were co-transfected into 293T cells at a 1:1 ratio, and the culture supernatants were collected 48 h after transfection. The supernatants were continuously passaged in donkey foetal dermal cells for three generations and the viral reverse transcriptase (RT) activities were determined using an RT assay kit (Roche, USA) according to the manufacturer’s instructions. The obtained recombinant EIAV strain was termed EIAV_MD. The 50% tissue culture infectious dose (TCID50) for each virus stock was determined in eMDM cells as described previously [1,19]. The diversity of each of these three diversity-varied strains was validated *in vitro* using high throughput sequence analysis of the *env* gene. The frequency of single nucleotide polymorphisms (SNPs) of *env* was separately calculated in the three *env* diversity-varied strains.

### Animal experiment design and evaluation

Clinical assessment was conducted using the challenge-immunosuppression protocol in three groups of five horses. Each group inoculated with one of the *env* diversity-varied EIAV strains. Each of these 15 horses was inoculated with 2 × 10^5^ TCID50 of the chosen viral strain. Another three horses serving as negative controls were “inoculated” with 2 ml sodium chloride saline. After inoculation, the temperature of each horse was daily monitored during the entire experiment. The number of platelets in peripheral blood was periodically measured on designated sampling days. At day 168 post-inoculation, each horse was challenged with the virulent EIAV_LN40_ strain (1 × 10^5^ TCID50). Post-challenge non-survivors were autopsied immediately after death. At 28 days post-challenge, all survivors were treated with daily administration of the immunosuppression agent dexamethasone at a dose of 0.11 mg/kg for 2 weeks [8,20]. At 14 days post-treatment with dexamethasone, suppressed immunity in the horses was confirmed using a Delayed Type Hypersensitivity (DTH) reaction on the skin and a cell proliferation assay on peripheral T cells via CFSE (5-carboxyfluorescein diacetate succinimidyl ester). All surviving horses with successful immunosuppression were monitored with continuing daily temperature measurements and periodical platelet measurements. At this point, all surviving horses were euthanized for autopsy and pathological scoring.

### Assessment of EIAV-host humoral immunity

Virus-specific humoral immunity was assessed using *env*-specific responses in sera between the three groups. Titres of *env*-specific antibodies (Abs) were measured using ELISA, as previously described [1]. The avidity of *env* antibody was measured by examining the stability of antigen/antibody complexes in the presence or absence of 6 M urea. The Avidity Index (AI = antibody activity treated with urea/untreated with urea) was used to calculate the avidity of *env*-specific antibodies as described previously [1]. Here the serum IgG antibody specific to Gag protein was chosen as a negative control. The quantitatively (*p26* point titre) and qualitatively (*p26* avidity index) were conducted using our standard ELISA procedures as previously described [19]. The activity of neutralizing antibodies against the challenging virus strain (EIAV_LN40_) or inoculating vaccine strain (EIAV_DLV121_) were determined using the luciferase-expressing virus reporter system. Briefly, EIAV-*env* packaged viruses (derived from the challenge strain and vaccine strain separately) were obtained through co-transfection with pONY8.1-Luc, pEIAV-GagPol, and the EIAV envelope expression plasmid. The supernatant was collected at 48h post-transfection. Subsequently, HEK293T/ELR1 cells were co-cultured using the supernatant with pseudo-virus mixed with inactivated sera for 48h. Pseudo-viruses were harvested to assess the activity of the neutralizing antibodies through the extent of inhibition of viral replication (as determined using a luciferase activity), using the following equation: % inhibition = {[luciferase activity with neutralizing antibodies/luciferase activity without neutralizing antibodies]} × 100. The background signal was determined using the sera from the healthy control.

### Quantitative assays for IFN-γ, GrzB and IL-2

The concentrations of three key virus-related cytokines (IFN**-γ**, GrzB and IL-2) in blood plasma were determined using a direct-ELISA method according to the manufacturer’s instructions (Bioaim Scientific, UK). The background signal was determined using the serum from the healthy group. Three independent experiments were performed for each treatment.

### Assessment of viral replication under host immunity

Plasma viral loads were analysed for the presence of viral RNA per millilitre of plasma by using a real-time RT–PCR assay based on *gag*-specific amplification primers [1,19]. The RNA copy numbers were determined by comparison with a standard curve obtained using known amounts of EIAV-*gag* RNA standards. A range of 10^2^–10^8^ copies of EIAV from *gag* standards was used as a reference. Data were collected and analysed during all stages of observation using the PE Applied Biosystems software.

### Isolation of nucleic acids and SNP calling using Illumina Hiseq

Isolation of nucleic acids and deep sequencing was performed using Illumina Hiseq. In brief, proviral DNA was extracted from eMDM cells *in vitro* that had been cultured and infected in parallel with either the EIAV_HD, EIAV_MD, or EIAV_LD (at the same TCID50) using the QIAamp DNA Blood Mini Kit (Qiagen, USA). The proviral DNA was amplified by PCR. Each PCR product was size-fractionated and recovered for sequencing (ligated with proprietary adaptors using T4 RNA ligase) according to the manufacturer’s instructions. The deep-sequencing experiment was performed at the Beijing Genomics Institute (Beijing, China), as a commercial service. Three different strains were sequenced separately using an Illumina Hiseq 2500 platform (Axeq Technologies, Rockville, MD, USA). An average depth of 150,000–210,000 reads for each nucleotide was generated. Sequence assembly and mapping were conducted on the 3500xL Genetic Analyzer.

### Assessment of clinical manifestations of disease and pathological changes in horses from autopsied samples

Clinical manifestations of disease in horses were individually assessed. Horse rectal temperature was recorded daily. Episodic fever was identified as being a temperature over 39°C. Horses with a platelet count in peripheral blood of less than 1 × 10^4^ per μl/blood were considered to have thrombocytopenia. Pathological assessments were performed on five autopsied organs by a professional pathologist. Autopsied specimens were embedded in paraffin for 24 h, then sliced and stained with haematoxylin and eosin (H&E) for morphological review. The five autopsied organs were lung, kidney, spleen, lymph nodes and liver. The severity of the pathological changes in each organ was categorized and scored independently between immunocompetent horses and immunosuppressed horses. For each organ, “0” indicated a normal organ, “1” indicated mild pathological changes, “2” indicated moderate pathological changes and “3” indicated severe pathological changes. A total of four professional pathologists scored each organ, by reviewing three randomly captured (×10 magnification) images independently under double-blind conditions. For each organ, the resultant score was the score assigned consistently by at least three pathologists. If no such consensus was reached, the organ was reassessed and scored by a panel of more than five pathologists.

### Statistical analyses

The *env* Abs titre, *env* Abs avidity and nAbs against the vaccine strain in the three different *env* diversity-varied groups of horses were compared using a linear mixed-effects model and adjusting for time. The group inoculated with the EIAV stain harbouring *env* moderate-diversity was taken as a reference. The package “lmerTest” in R (Version 3.6.0) was used to carry out the estimation. Multivariate ANOVA inference was employed to test for significance between the different characteristics of the three *env* diversity-varied EIAV strains. Results were considered significant or very significant when *P-*values were less than 0.05 or 0.01, respectively.

## Results

### Construction and verification of three *env* diversity-varied EIAV strains *in vitro*

**Figure 1.**
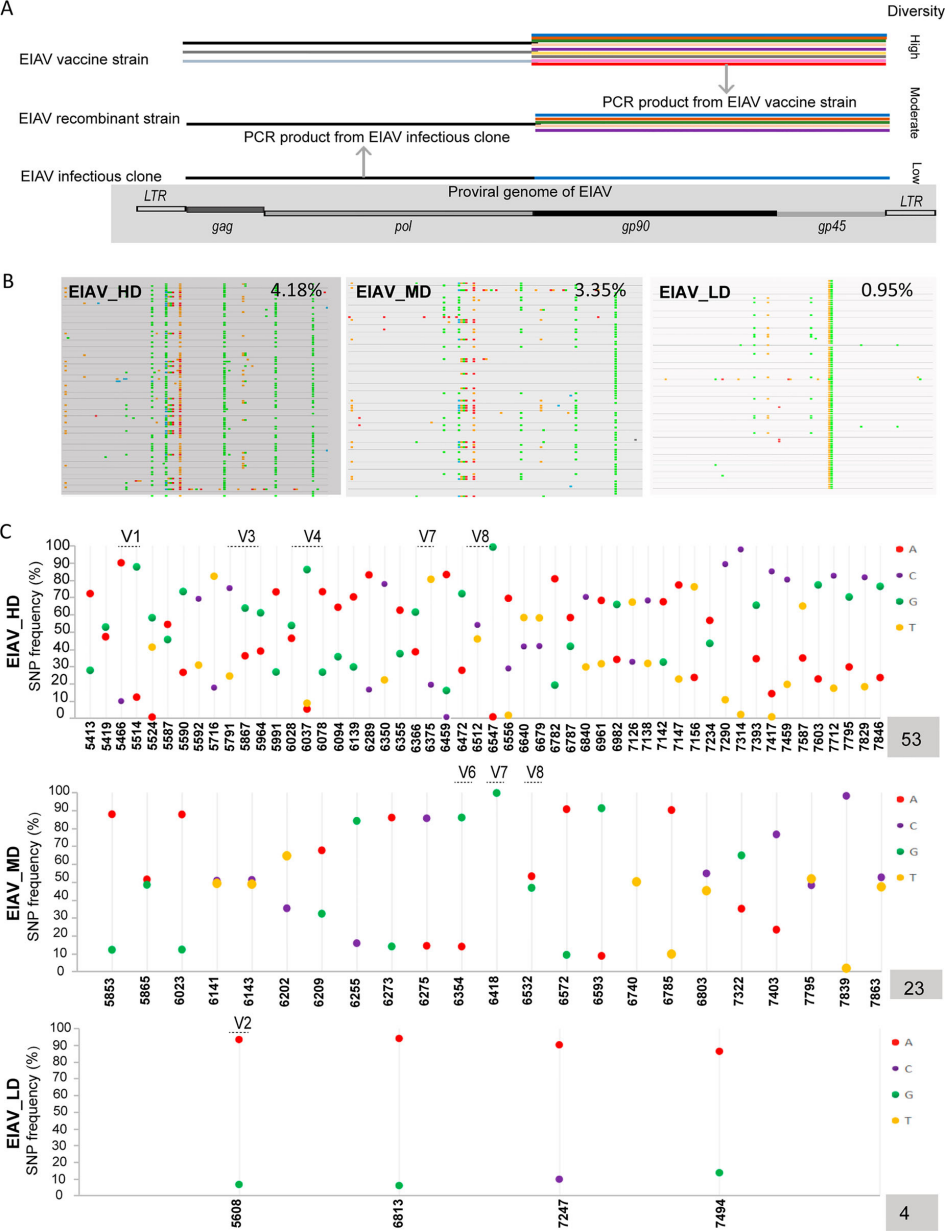
Construction of the three EIAV *env* diversity-varied virus candidates. (A) Schematics of diversity-varied EIAV strains. An EIAV vaccine strain represented high-diversity EIAV (EIAV_HD) whereas an EIAV infectious clone represented low-diversity EIAV (EIAV_LD). A homologous recombinant EIAV strain combining a *gag-pol* PCR product from the EIAV infectious clone with a PCR product from the EIAV vaccine was obtained to represent the moderate-diversity EIAV (EIAV_MD). (B) Three strains with supposedly varied *env-*diversity were validated by the distribution of *env* nucleotides using Molecular Evolutionary Genetics Analysis (MEGA) analysis. Different types of nucleotides are represented with different colours with distinct genetic distances. (C) The number of SNPs on *env* was estimated using a deep sequencing technique to confirm the variation of *env-*diversity.

Previous research using sequencing techniques revealed that the diversity of the attenuated EIAV vaccine was four times higher than its parent strain (Figure S1). Furthermore, a clear parallel was found between the increased diversity and the increased passaging *in vitro* during the developing attenuation of virulent strain [10]. Here, three EIAV strains supposedly varying in *env* diversity were constructed, including an attenuated vaccine EIAV_HD, a recombinant strain EIAV_MD, and a monoclonal infectious clone EIAV_LD (Figure 1(A)) (details are given in materials and methods) [17]. We compared the frequency of mutated sites in the three strains by conventional PCR and sequencing. The results showed that the frequency of mutation of EIAV_MD was intermittent between that of EIAV_HD and that of EIAV_LD (Figure 1(B)). We further confirmed this result by studying highly informative SNP through deep sequencing, and compared the numbers of highly mutated sites on *env* in the three vaccine candidates. In EIAV_HD characterized by high *env* diversity, there were five highly variable regions (V1/V3/V4/V7/V8). In EIAV_MD, three highly variable regions (V6/V7/V8) were seen. Only a single highly variable region (V2) was found in EIAV_LD (Figure 1(C)). Simultaneously, western blotting, RT activity assays and real-time RT PCR were performed to ensure that all viruses were correctly expressed and harvested at a comparable level. Hence, we have obtained three vaccine candidates with gradually reduced diversity in their *env* genes *in vitro*.

### Clinical manifestation of disease in horses vaccinated with the vaccine candidates before and after viral challenge

**Figure 2.**
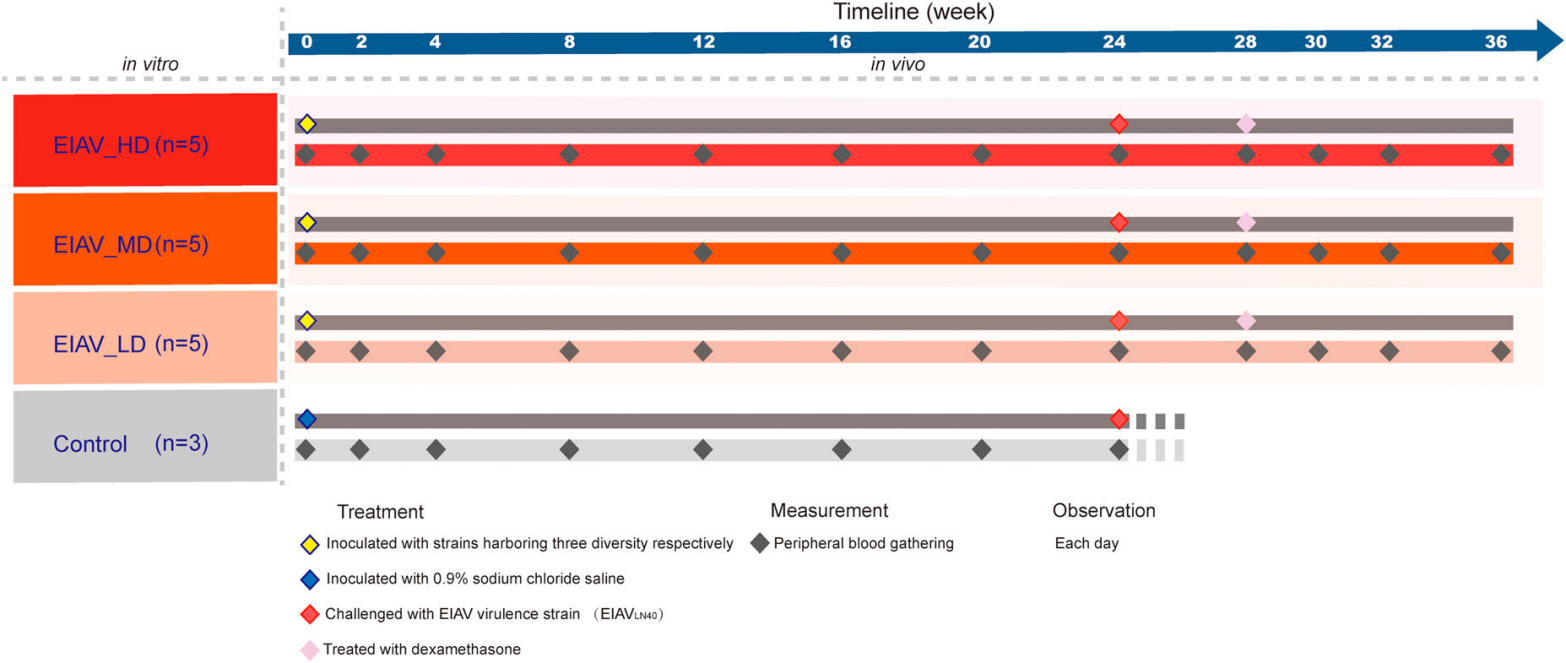
The timeline of treatment and periodical measurement on experimental horses undergoing inoculation with *env* diversity-varied EIAV strains *in vivo*. The three *env* diversity-varied EIAV strains are represented by the three brown colour gradients. Fifteen experimental horses were randomly divided into three groups and were inoculated with three *env* diversity-varied EIAV strains respectively. Another three horses (light-grey colour) were chosen as sham control. Within each inoculated group, inoculation (day 0), challenge (24 weeks) and immunosuppression (28 weeks) are dotted along the upper line. Administration of dexamethasone for 14 days could inhibit the host immunity continuously to reach an immunosuppressed status. Peripheral blood was sampled approximately every two weeks in survivors.

**Figure 3.**
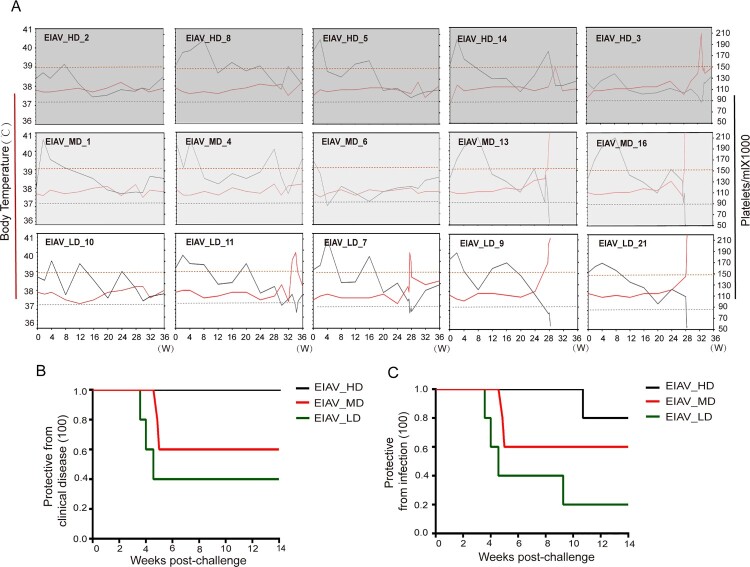
EIA clinical manifestation and death in 15 horses inoculated with 3 different *env* diversity-varied strains. (A) Individual-based daily clinical records were shown cross three inoculated groups including with EIAV_HD (upper line, background with the darkest grey gradient), EIAV_MD (middle line, background with the moderately dark gradient) and EIAV_LD (lower line, background with the lightest grey gradient). Rectal temperature greater than 39°C (red dotted line) and/or platelets in peripheral blood of 100,000/μl (blue dotted line) resulted in a diagnosis of EIA cross the three groups. (B) Kaplan-Meier curve of EIA protection conferred by the three *env* diversity-varied strains. Kaplan-Meier curves on EIA proportion post-challenge between three groups have implied the protective potential of three *env* diversity-varied EIAVs. Notably, the left Kaplan-Meier curve depicts the EIA-to-time before immunosuppression whereas the right Kaplan-Meier curve depicts the real EIA-to-time from prior to post immunosuppression. The left-right curves comparison has further exhibited the diversity-dependent protection post-challenge (left) being “amplified” under immunosuppression (right).

Fifteen horses were randomly divided into three groups and inoculated with indicated vaccine candidates, with an independent control group of three horses inoculated with saline buffer, as indicated in the schematic diagram (Figure 2). All the animals were challenged with a virulent strain EIAV_LN40_ at 24 weeks post-inoculation. The surviving horses were subjected to a follow-up treatment of dexamethasone to mimic an immunosuppression stage. The timeline of the whole experiment can be broken down into three stages: a 24-week stage post-inoculation and prior to challenge; a 4-week stage post-challenge but prior to immunosuppression, and an 8-week stage post immunosuppression. The immunity of the host can therefore be considered as immunocompetent (week 0–24) followed with immunosuppression (week 28–30). The rectal temperature was monitored and the number of platelets in peripheral blood was assessed throughout the experiment (Figure 3(A)). All the animals lived with normal health conditions in the first stage of 24 weeks and have generally similar viral load before challenge (2 × 10^2^ to 4 × 10^3^ copies/ml). There was insignificant variation in the viral load at different time points in sera from horses inoculated with the three *env* diversity-varied EIAV strains (Figure S2). After challenge, all the horses in the control group, 2 horses from the EIAV_LD group, and 2 horses from the EIAV_MD group developed acute disease that resulted in death. One horse in the EIAV_LD group developed acute EIA, with concurrent fever and marked decline in platelets, but did not die. The viral load in all the horses that developed clinical symptoms reached high levels (>1 × 10^7^ copies/ml). However, none of the horses inoculated with the high-diversity vaccine developed disease post-challenge and prior to immunosuppression, indicating that the EIAV_HD provided complete protection. Moreover, the horses without any clinical EIA symptoms harboured a low viral load (2 × 10^2^–4 × 10^3^ copies/ml) (Figure S2). The three mock-inoculated (sham control) horses displayed acute EIAs and died within 28 days post-challenge, and all had viral loads above 1 × 10^7^ copies/ml (Figure S2).

High fever and low levels of platelets were associated with the development of EIAs in certain animals after challenge (Figure 3(A)). None of the animals had fever before challenge (week 24). All the animals in EIAV_HD group, 60% animals in EIAV_MD group, and 40% animals in EIAV_LD group were clinically protected from EIA (Figure 3(B)). In order to confirm the protection of infection against EIAV, horses were subjected to immunosuppression to suppress the host immunity and to exacerbate infection. At the post-immunosuppression stage, one horse inoculated in the EIAV_HD group developed EIA, but without death. Another horse inoculated with EIAV_LD also displayed a similar outcome. If we consider that possible outcomes include EIA with or without death, the group inoculated with EIAV_HD was 80% protected from EIAV infection, whereas the protective efficacy was reduced to 20% for the group inoculated with EIAV_LD. In the group inoculated with EIAV_MD with moderate *env* diversity, 60% of horses were protected (Figure 3(C)). These results clearly reveal a critical correlation between complexity of *env* and protective efficacy.

### Comparison of humoral immunity between three groups inoculated with *env* diversity-varied EIAV strains

**Figure 4.**
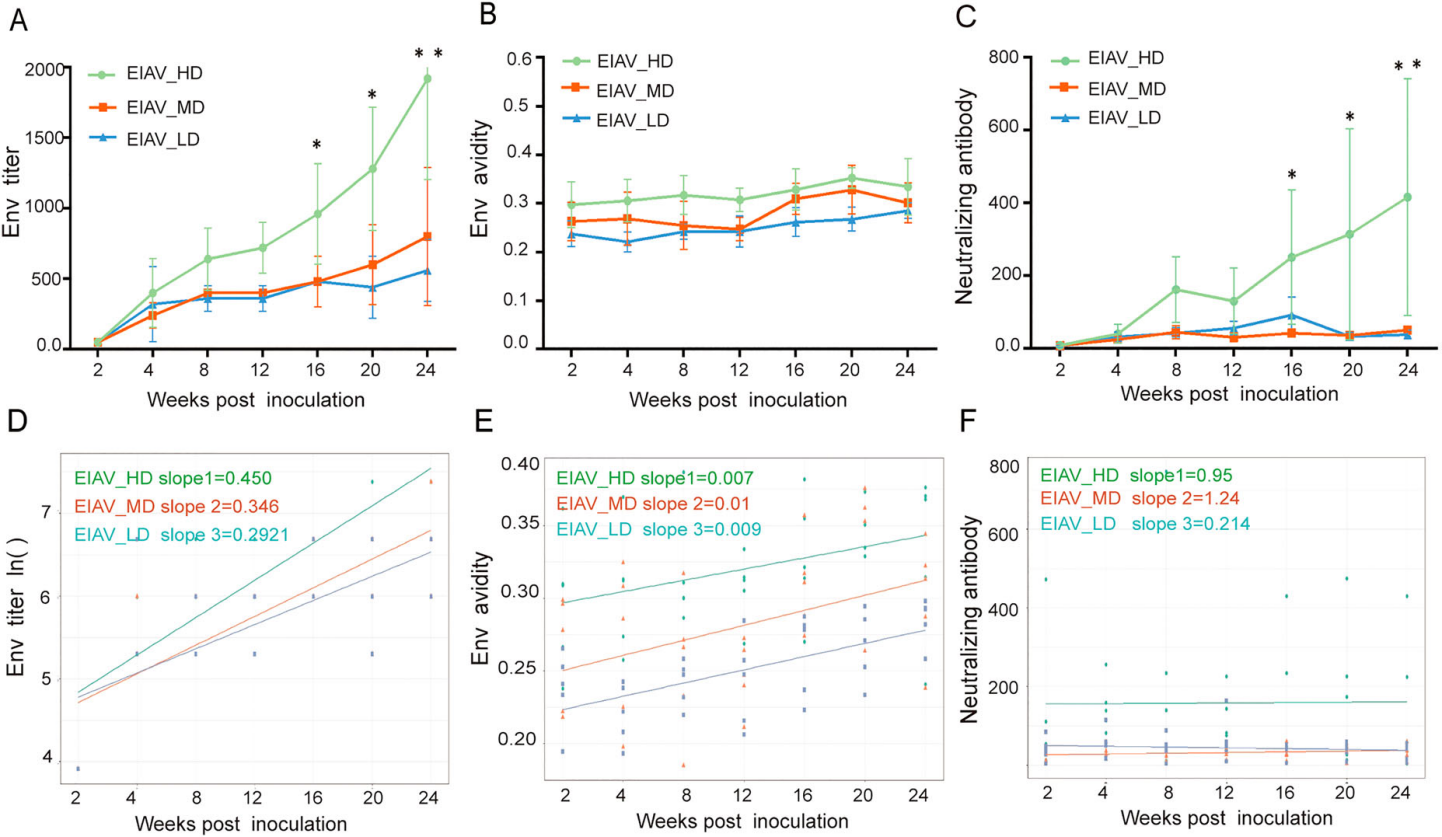
Time-dependent trajectories of *env* antibody titre, *env* antibody avidity and neutralizing antibody against vaccine strain in three diversity-dependent inoculated groups. (A–C) The time-dependent titre of *env* Abs, *env* Abs avidity and nAbs against vaccine strain are shown cross three inoculated groups. (D–F) The time-dependent slopes of the *env* Abs titre, *env* Abs avidity and the titre of nAbs were compared between three groups based on supposed linear regression. The steepest slope by time was seen in the group inoculated with EIAV_HD.

The humoral responses to EIAV in all vaccinated horses were evaluated by detection and analysis of the titre of *env*-specific Abs (Figure 4(A)), the avidity of *env*-specific Abs (Figure 4(B)), and the titre of nAbs against EIAV vaccine strain (Figure 4(C)) from the time of the inoculation to the time of challenge. The temporal profile showed that the titres of *env*-specific Abs and nAbs against EIAV vaccine strain in the EIAV_HD group were significantly higher compared to the other two groups, especially at 16 weeks post inoculation (Figure 4(A,C)). Apart from the fact that a gradual increase is seen in the titre of *env* Abs, and the avidity and the titre of nAbs in all groups, the slopes and expression levels are different. A linear-like correlation can be seen between the humoral immune response and the *env* diversity of the inoculating EIAV strain. Using trajectories based on linear regression (Figure 4(D–F)), a significantly higher and faster increasing *env*-specific Ab was observed in the group inoculated with EIAV_HD compared to the other two groups (*P *< 0.05). Similarly increasing speed of avidity or nAbs were seen in all three groups inoculated with diversity-varied strains. However, the magnitude of avidity displayed a diversity-dependent gradient. Additionally, the diversity-non-dependent control, the titre and avidity of *gag*-specific Abs showed no discrepancy in three groups. Only at 4 weeks post inoculation were the titres of *gag* (p26) significantly greater in EIAV_LD than in the other groups (Figure S3). Using a linear mixed effects model with adjustment for time, this disparity can be clearly demonstrated (Table 1). Taking the group inoculated with EIAV_MD as a reference, a significant enhancement in *env* Abs titre, *env* Abs avidity and nAbs against vaccine strain could be seen in all five horses inoculated with the EIAV_HD (all *P *< 0.01). In the group inoculated with EIAV_LD, only the *env* antibody avidity was significantly lower than the reference group, indicating that EIAV_LD could arouse similar humoral immunity to EIAV_MD strain. We also analysed correlations between *env* diversity and nAbs to the challenge strain and several immune cytokines (IFN-γ, IL-2 and GzmB) pre /post-challenge. Although the nAbs against the challenge strain were found to be low during the observation period, a milder disparity was also observed between the three groups pre and post-challenge (Table S1). Moreover, a significant correlation could be seen between SNPs and nAbs for the challenge strain and IFN-γ prior and post-challenge using a Pearson method. (*P *< 0.05) (Table S2). A significant correlation could be seen between SNPs and nAbs against the challenge strain and IFN-γ prior and post-challenge (*P* < 0.05) (Table S2). Only a mild correlation between SNPs and IL-2 was seen post-challenge. The enhanced nAbs and IFN-γ observed might be elicited by viral strains with increased diversity. In summary, higher diversity of the EIAV strain elicited greater humoral immunity even for IFN-γ than in lower diversity EIAV strains.

**Table 1.** Comparison of *env* Abs titre, *env* Abs avidity and nAbs against vaccine strain from the horses inoculated with three env diversity-varied strains, as calculated using a linear mixed effects model.

*env* diversity	titre of *env*-specific Abs		avidity of *env*-specific Abs		nAbs against vaccine strain	
	*T* value	*P-*value	*T* value	*P-*value	*T* value	*P-*value
EIAV_MD (Ref)						
EIAV_HD	3.367	0.001	2.994	0.009	4.92	3.19E-06
EIAV_MD (Ref)						
EIAV_LD	−0.5398	0.591	−2.367	0.032	0.518	0.605

Note: A linear mixed effects model was used here to compare three indicators (*env* Abs titre, *env* Abs avidity and nAbs against the vaccine strain) in the different *env* diversity-varied groups of horses. *P* < 0.01 indicated statistical significance.

### High *env*-diversity vaccine provided better protection from infection and pathological damage

**Figure 5.**
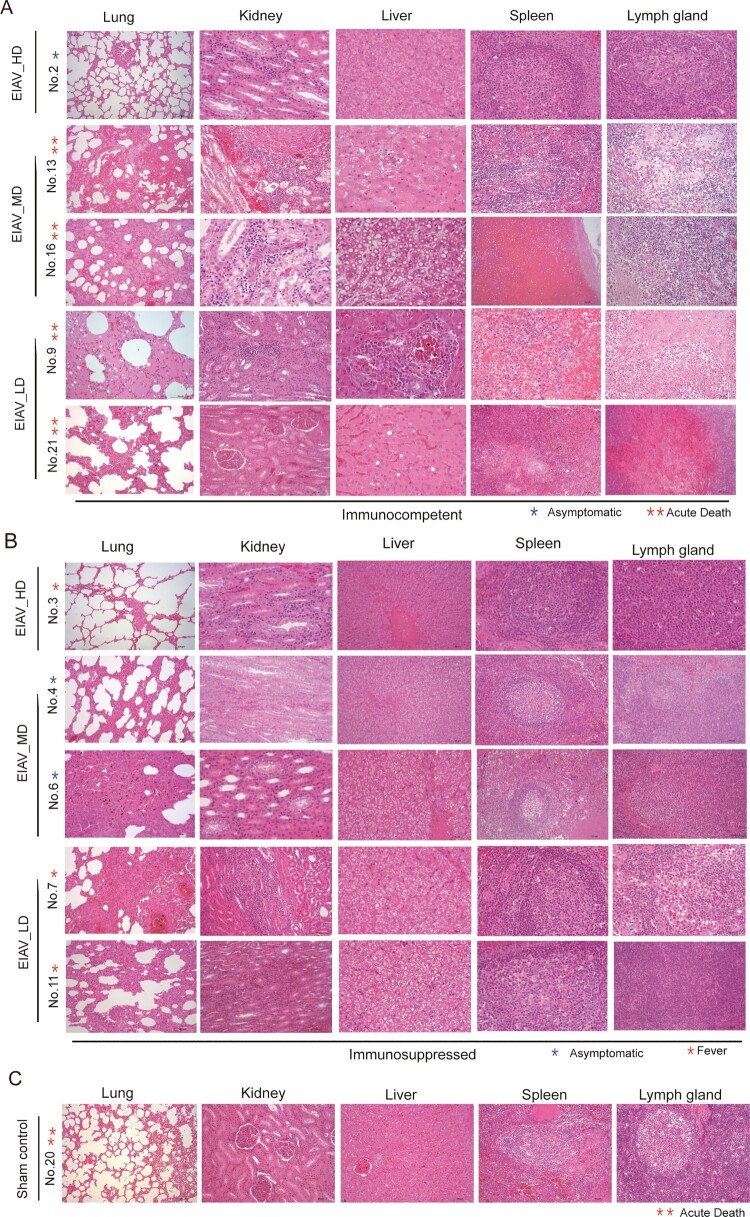
Comparison of pathological changes in specimens from immunocompetent horses (A), immunosuppressed horses (B) and control horses (C). The typical pathological changes observed in lung, kidney, liver, spleen and lymph gland are presented on pictures separately (haematoxilin and eosin 4 m paraffin sections, original magnification 10×). Pathological lesions severity and scoring are given on Table 2. All organs and tissues are from horses developed EIA clinical symptoms (except EIAV_HD_2) prior and after immunosuppressed stage.

We next examined the pathological changes in tissues from horses that had either died from the disease or had been euthanized after challenge with a virulent viral strain. At the immunocompetent stage, four horses, two from EIAV_MD group (No.13 and NO.16) and two from EIAV_LD group (No.9 and No. 21), developed acute EIA and died post-challenge. The pathological scores given to the organs of these horses after autopsy are shown in Figure 5(A). As all horses from the EIAV_HD group survived, one horse from this group was euthanized for in-parallel comparison. The remaining 10 vaccinated horses that survived until the end of experiment underwent euthanasia for pathological studies.

An inflammation varying in extent could be seen in different tissues from the three *env* diversity-varied groups separately. In general, pathological changes were much milder in specimens from immunosuppressed horses than those from immunocompetent horses. For example, the changes seen in the lungs included sparse or abundant oedema with congested capillaries and dilated alveoli with widened alveolar septa. In the spleen, horses underwent detectable structure disarrangement with mild fibrosis. In the lymphoid tissue, mild-to-moderate chronic adaptive changes, including hyaline degeneration and interstitial fibrosis, were seen in organs (Figure 5(A,B)). In contrast, the typical and severe pathological changes were seen in different tissues of the horses from the control group after challenge with the virulence EIAV strain (Figure 5(C)). In the five immunocompetent horses, a mild parallel could be seen between the severity of the pathological damage and the diversity of EIAV stain with which the horse had been inoculated (Table 2). In the lungs and kidneys, immunopathological lesions diffused in interstitial tissue were inversely proportional to the magnitude of *env*-diversity. In the liver and lymphoid tissues, the extent of chronic lesions was moderately inversely correlated with the *env* diversity of the inoculating strain. One horse from the EIAV_MD group exhibited unexpectedly severe lesions in the spleen. Although the severity of pathological lesions was generally much milder after immunosuppression, which is consistent with milder clinical EIA, the effect of *env-*diversity was always visible (Table 2). The pathological changes observed in the organs from the immunosuppressed survivors showed mild differences between the three diversity-dependent groups. These pathological results provided further evidence of *env* diversity-dependent protection against EIAV infection.

**Table 2.** Semi-quantitative scores of pathological lesions on five autopsied organs from challenged horses inoculated with three env diversity-varied strains, prior and post immunosuppressed.

Stage	Group	Animal ID	Pathological changes score				
			lung	liver	kidney	spleen	Lymph node
Immunocompetent	EIAV_HD	2	0	2	1	0	0
	EIAV_MD	13	2	1	2	2	1
	EIAV_LD	16	2	2	2	3	1
	EIAV_LD	9	3	1	1	3	3
	EIAV_LD	21	3	2	3	2	2
Immunosuppressed	EIAV_HD	3	0	0	1	0	0
	EIAV_MD	4	0	0	1	0	0
	EIAV_LD	6	0	0	0	1.5	0
	EIAV_LD	7	0.5	0	0.5	1.5	1
	EIAV_LD	11	0	0.5	0.5	0.5	0.5

Note: Semi-quantitative scoring method was used to reflect the severity of organs from five immunocompetent horses and five immunosuppressed horses. “0” indicated a normal organ, “1” indicated mild pathological changes, “2” indicated moderate pathological changes and “3” indicated severe pathological changes.

## Discussion

Enhanced immunogenic diversity has contributed substantially to the successful development of vaccines, especially against lentiviruses [21–23]. To obtain diversity in vaccines, numerous approaches, including sequential immunization, centralized or “mosaic” immunogens have been continually developed for protection against HIV infection [15,24–26]. However, to date, lentiviral vaccines against highly varied HIV-1 strains lack robust efficacy [27,28]. Eliciting broad protection against infection therefore remains a challenge [22,23,25,29–32].

We hypothesized that a diversity-dependent mechanism should exist during virus-host interactions. We used a well-characterized EIAV attenuated vaccine able to provide effective immune protection against a heterogenous virulence strain, and have constructed a diversity-dependent platform to verify our hypothesis. Our findings have confirmed our hypothesis that the *env* diversity of the immunogens is able to robustly protect the host against EIAV infection. We observed a remarkable *env-*diversity-dependent protective disparity between the EIAV_HD (the EIAV attenuated vaccine with high *env* diversity), EIAV_MD (a recombinant strain with moderate *env* diversity) and EIAV_LD (an infectious clone with low *env* diversity). Statistical analysis has revealed a linear correlation between the extent of *env-*diversity and protection against challenging EIAV infection. The immunosuppression of hosts inoculated with diversity-varied stains has further “highlighted” the existing linear correlation between *env*-diversity and protection. We supposed that the suppression of host immunity could enhance the viral replication, which was validated by the replicating curve prior and post-challenge. Combining the results of three *env* diversity-varied inoculated groups prior to challenge and post-challenge as well as prior to immunosuppression and post-immunosuppression, it is clear that *env* diversity plays an important role in immune protection against EIAV. To the best of our knowledge, this is the first study demonstrating how *env* diversity-varied lentiviral vaccines protected hosts against viruses, leaving behind decades of Gag being used as a primary target immunogen [33–36].

To better understand the mechanism of *env* diversity-dependent protection, *env*-specific Abs were periodically measured and compared between groups. As expected, there was a linear correlation between the titre, avidity of *env*-specific Abs or nAbs against vaccine, and the magnitude of *env-*diversity of the three inoculated strains. These results suggest that increased *env-*diversity has a protective mechanism as it enhances humoral immunity through both eliciting and maturing *env*-specific Abs. Notably, the titre of nAb in HD group is much higher than the other groups. Unexpectedly, we observed a markedly low titre of nAbs in EIAV_MD group, similar to that of the EIAV_LD group. By reviewing the deep sequencing database and searching for differences between strains, we found that a hypervariable region V3, which is correlated with neutralization antibody response [37,38], is absent in EIAV_MD sequences. It has been previously documented that the hypervariable V3 region within vaccine EIAV is capable to elicit an overwhelming abundance of nAbs. It is very likely that the V3 region was excluded during the random homogenous recombination process used to generate EIAV_MD, leading to a significantly lower titre of neutralizing antibodies in EIAV_MD than in EIAV_HD. Regions with high *env*-diversity could also play a role in autologous immune escape, which has been substantially documented in HIV-1 [15,39]. Despite the formidable challenge of designing vaccines targeting variable regions in *env*, it is nevertheless of great importance that we continue to attempt this, in order to be able to target the hypervariable regions and wider epitopes from the diverse quasispecies that may exist in viral stocks [40,41]. Here we adopted a recombinant PCR method based on a dominance-based amplification rationale in pooled variants and microvariants of the viral population to mimic the vaccine diversity. We recommended our platform to any lentivirus researchers attempting to mimic natural diversity in lentiviruses.

In general, combining results from both our *in vitro* and *in vivo* studies, the data presented here using EIAV *env* diversity-varied viruses clearly demonstrate the effects of *env* diversity on vaccine efficacy. Moreover, the complexity of the *env* diversity was able to increase during the virus-host interactions. Numerous HIV vaccines developed with enhanced diversity have conferred higher protection compared to the low diverse controls [21,22,42,43]. Notably, both the diversity region and the extent of the diversity were important during the development of the attenuated EIAV vaccine. Moreover, this diversity of immunogen-based attenuated vaccine is likely to need to incorporate strategies to expand the *env*-specific Abs precursor pool. In addition, we found that the significance of *env* diversity for vaccine efficacy does not preclude a role for *gag* diversity in a protective vaccine. Therefore, additional studies are still required for specific validation, and there is still a long way to go before potent lentivirus vaccines can be reliably developed. We believe that our observations using *env* diversity-varied strains could provide useful information for the design of vaccine regimens for the other members of the lentivirus family.

## Supplementary Material

Table_S1_and_S2.docx

## References

[1] Lin YZ, Shen RX, Zhu ZY, et al. An attenuated EIAV vaccine strain induces significantly different immune responses from its pathogenic parental strain although with similar in vivo replication pattern. Antiviral Res. 2011 Nov;92(2):292–304. 21893100 10.1016/j.antiviral.2011.08.016

[2] Wang XF, Liu Q, Wang YH, et al. Characterization of equine infectious anemia virus long terminal repeat quasispecies *in vitro* and *in vivo*. J Virol. 2018 Apr 15;92(8). doi: e02150-17 10.1128/JVI.02150-17 PMC5874411 29386282

[3] Wang X, Wang S, Lin Y, et al. Genomic comparison between attenuated Chinese equine infectious anemia virus vaccine strains and their parental virulent strains. Arch Virol. 2011 Feb;156(2):353–357. 21136127 10.1007/s00705-010-0877-8

[4] Wang XF, Lin YZ, Li Q, et al. Genetic Evolution during the development of an attenuated EIAV vaccine. Retrovirology. 2016 Feb 3;13:9. 26842878 10.1186/s12977-016-0240-6 PMC4738788

[5] Wei X, Decker JM, Wang S, et al. Antibody neutralization and escape by HIV-1. Nature. 2003 Mar 20;422(6929):307–312. 12646921 10.1038/nature01470

[6] Sekaly RP. The failed HIV Merck vaccine study: a step back or a launching point for future vaccine development? J Exp Med. 2008 Jan 21;205(1):7–12. 18195078 10.1084/jem.20072681 PMC2234358

[7] Chen Z. Monkey models and HIV vaccine research. Adv Exp Med Biol. 2018;1075:97–124. 30030791 10.1007/978-981-13-0484-2_5

[8] Craigo JK, Zhang B, Barnes S, et al. Envelope variation as a primary determinant of lentiviral vaccine efficacy. Proc Natl Acad Sci U S A. 2007 Sep 18;104(38):15105–15110. 17846425 10.1073/pnas.0706449104 PMC1986620

[9] Bell SM, Bedford T. Modern-day SIV viral diversity generated by extensive recombination and cross-species transmission. Plos Pathog. 2017 Jul;13(7):e1006466. 28672035 10.1371/journal.ppat.1006466 PMC5510905

[10] Liu C, Wang XF, Wang Y, et al. Characterization of EIAV env quasispecies during long-term passage in vitro: gradual loss of pathogenicity. Viruses. 2019 Apr 24;11(4):380. 10.3390/v11040380 PMC6520696 31022927

[11] Tagmyer TL, Craigo JK, Cook SJ, et al. Envelope determinants of equine infectious anemia virus vaccine protection and the effects of sequence variation on immune recognition. J Virol. 2008 Apr;82(8):4052–4063. 18234792 10.1128/JVI.02028-07 PMC2292999

[12] Moore PL. The neutralizing antibody response to the HIV-1 Env protein. Curr HIV Res. 2018;16(1):21–28. 29173180 10.2174/1570162X15666171124122044 PMC6234226

[13] Steinhardt JJ, Guenaga J, Turner HL, et al. Rational design of a trispecific antibody targeting the HIV-1 Env with elevated anti-viral activity. Nat Commun. 2018 Feb 28;9(1):877. 29491415 10.1038/s41467-018-03335-4 PMC5830440

[14] Seaman MS, Xu L, Beaudry K, et al. Multiclade human immunodeficiency virus type 1 envelope immunogens elicit broad cellular and humoral immunity in rhesus monkeys. J Virol. 2005 Mar;79(5):2956–2963. 15709015 10.1128/JVI.79.5.2956-2963.2005 PMC548456

[15] Sadanand S, Suscovich TJ, Alter G. Broadly neutralizing antibodies against HIV: new insights to inform vaccine design. Annu Rev Med. 2016;67:185–200. 26565674 10.1146/annurev-med-091014-090749

[16] Zhu C, Dukhovlinova E, Council O, et al. Rationally designed carbohydrate-occluded epitopes elicit HIV-1 Env-specific antibodies. Nat Commun. 2019 Feb 27;10(1):948. 30814513 10.1038/s41467-019-08876-w PMC6393580

[17] Wang XF, Bai B, Lin Y, et al. High-efficiency rescue of equine infectious anemia virus from a CMV-driven infectious clone. Virol Sin. 2019 Dec;34(6):725–728. 31376080 10.1007/s12250-019-00153-w PMC6888787

[18] Braun MJ, Clements JE, Gonda MA. The visna virus genome: evidence for a hypervariable site in the env gene and sequence homology among lentivirus envelope proteins. J Virol. 1987 Dec;61(12):4046–4054. 2824836 10.1128/jvi.61.12.4046-4054.1987 PMC256031

[19] Lin YZ, Cao XZ, Li L, et al. The pathogenic and vaccine strains of equine infectious anemia virus differentially induce cytokine and chemokine expression and apoptosis in macrophages. Virus Res. 2011 Sep;160(1–2):274–282. 21782860 10.1016/j.virusres.2011.06.028

[20] Ma J, Shi N, Jiang CG, et al. A proviral derivative from a reference attenuated EIAV vaccine strain failed to elicit protective immunity. Virology. 2011 Feb 5;410(1):96–106. 21094511 10.1016/j.virol.2010.10.032

[21] Mann JK, Ndung’u T. HIV-1 vaccine immunogen design strategies. Virol J. 2015 Jan 24;12:3. 25616599 10.1186/s12985-014-0221-0 PMC4318220

[22] Korber B, Hraber P, Wagh K, et al. Polyvalent vaccine approaches to combat HIV-1 diversity. Immunol Rev. 2017 Jan;275(1):230–244. 28133800 10.1111/imr.12516 PMC5362114

[23] Hurwitz JL, Bonsignori M. Multi-envelope HIV-1 vaccine development: two targeted immune pathways, one desired protective outcome. Viral Immunol. 2018 Mar;31(2):124–132. 29315059 10.1089/vim.2017.0144 PMC5915263

[24] Badamchi-Zadeh A, McKay PF, Korber BT, et al. A multi-component prime-boost vaccination regimen with a consensus MOMP antigen enhances Chlamydia trachomatis clearance. Front Immunol. 2016;7:162. 27199987 10.3389/fimmu.2016.00162 PMC4848310

[25] Kamlangdee A, Kingstad-Bakke B, Osorio JE. Mosaic H5 Hemagglutinin provides broad humoral and cellular immune responses against influenza viruses. J Virol. 2016 Aug 1;90(15):6771–6783. 27194759 10.1128/JVI.00730-16 PMC4944288

[26] Escolano A, Dosenovic P, Nussenzweig MC. Progress toward active or passive HIV-1 vaccination. J Exp Med. 2017 Jan;214(1):3–16. 28003309 10.1084/jem.20161765 PMC5206506

[27] McGuire AT, Hoot S, Dreyer AM, et al. Engineering HIV envelope protein to activate germline B cell receptors of broadly neutralizing anti-CD4 binding site antibodies. J Exp Med. 2013 Apr 8;210(4):655–663. 23530120 10.1084/jem.20122824 PMC3620356

[28] Sliepen K, Sanders RW. HIV-1 envelope glycoprotein immunogens to induce broadly neutralizing antibodies. Expert Rev Vaccines. 2016;15(3):349–365. 26654478 10.1586/14760584.2016.1129905

[29] Wang S, Pal R, Mascola JR, et al. Polyvalent HIV-1 Env vaccine formulations delivered by the DNA priming plus protein boosting approach are effective in generating neutralizing antibodies against primary human immunodeficiency virus type 1 isolates from subtypes A, B, C, D and E. Virology. 2006 Jun 20;350(1):34–47. 16616287 10.1016/j.virol.2006.02.032

[30] Abdul-Jawad S, Ondondo B, van Hateren A, et al. Increased valency of conserved-mosaic vaccines enhances the breadth and depth of Epitope recognition. Mol Ther. 2016 Feb;24(2):375–384. 26581160 10.1038/mt.2015.210 PMC4817818

[31] Cai H, Zhang R, Orwenyo J, et al. Multivalent antigen presentation enhances the immunogenicity of a synthetic three-component HIV-1 V3 Glycopeptide vaccine. ACS Cent Sci. 2018 May 23;4(5):582–589. 29806004 10.1021/acscentsci.8b00060 PMC5968512

[32] Xu K, Acharya P, Kong R, et al. Epitope-based vaccine design yields fusion peptide-directed antibodies that neutralize diverse strains of HIV-1. Nat Med. 2018 Jun;24(6):857–867. 29867235 10.1038/s41591-018-0042-6 PMC6358635

[33] Xu F, Hong M, Ulmer JB. Immunogenicity of an HIV-1 gag DNA vaccine carried by attenuated Shigella. Vaccine. 2003 Jan 30;21(7-8):644–648. 12531333 10.1016/s0264-410x(02)00573-x

[34] Jiang WZ, Jin NY, Li ZJ, et al. [Study on the immunogenicity of HIV-1 gag vaccine]. Xi Bao Yu Fen Zi Mian Yi Xue Za Zhi. 2004 May;20(3):272–273. 15193215

[35] Betts MR, Exley B, Price DA, et al. Characterization of functional and phenotypic changes in anti-Gag vaccine-induced T cell responses and their role in protection after HIV-1 infection. Proc Natl Acad Sci USA. 2005 Mar 22;102(12):4512–4517. 15753288 10.1073/pnas.0408773102 PMC552973

[36] Breton M, Zhao C, Ouellette M, et al. A recombinant non-pathogenic Leishmania vaccine expressing human immunodeficiency virus 1 (HIV-1) Gag elicits cell-mediated immunity in mice and decreases HIV-1 replication in human tonsillar tissue following exposure to HIV-1 infection. J Gen Virol. 2007 Jan;88(Pt 1):217–225. 17170454 10.1099/vir.0.81995-0

[37] Leroux C, Issel CJ, Montelaro RC. Novel and dynamic evolution of equine infectious anemia virus genomic quasispecies associated with sequential disease cycles in an experimentally infected pony. J Virol. 1997 Dec;71(12):9627–9639. 9371627 10.1128/jvi.71.12.9627-9639.1997 PMC230271

[38] Howe L, Leroux C, Issel CJ, et al. Equine infectious anemia virus envelope evolution in vivo during persistent infection progressively increases resistance to in vitro serum antibody neutralization as a dominant phenotype. J Virol. 2002 Nov;76(21):10588–10597. 12368301 10.1128/JVI.76.21.10588-10597.2002 PMC136617

[39] Zhou T, Georgiev I, Wu X, et al. Structural basis for broad and potent neutralization of HIV-1 by antibody VRC01. Science. 2010 Aug 13;329(5993):811–817. 20616231 10.1126/science.1192819 PMC2981354

[40] Posch PE, Araujo HA, Creswell K, et al. Microvariation creates significant functional differences in the DR3 molecules. Hum Immunol. 1995 Jan;42(1):61–71. 7751161 10.1016/0198-8859(94)00074-z

[41] Fujita Y, Otsuki H, Watanabe Y, et al. Generation of a replication-competent chimeric simian-human immunodeficiency virus carrying env from subtype C clinical isolate through intracellular homologous recombination. Virology. 2013 Feb 5;436(1):100–111. 23219366 10.1016/j.virol.2012.10.036

[42] Roederer M, Keele BF, Schmidt SD, et al. Immunological and virological mechanisms of vaccine-mediated protection against SIV and HIV. Nature. 2014 Jan 23;505(7484):502–508. 24352234 10.1038/nature12893 PMC3946913

[43] Kim JH, Excler JL, Michael NL. Lessons from the RV144 Thai phase III HIV-1 vaccine trial and the search for correlates of protection. Annu Rev Med. 2015;66:423–437. 25341006 10.1146/annurev-med-052912-123749

