# Supplementary material for: Env diversity-dependent protection of the attenuated equine infectious anaemia virus vaccine
Source: Emerg Microbes Infect. 2020 Jun 11;9(1):1309–20. doi: 10.1080/22221751.2020.1773323 (PMC7473056; doi:10.1080/22221751.2020.1773323)
Supplement: Table_S1_and_S2.docx [file TEMI_A_1773323_SM0941.docx]

| **Group** | **Animal ID** |  | **Clinical outcome** | | |  | **6+ month before challenge** | | | |  | **0.5+ month after challenge** | | | | |
| --- | --- | --- | --- | --- | --- | --- | --- | --- | --- | --- | --- | --- | --- | --- | --- | --- |
|  |  |  | **Fever** | **Virus load** | **Death** |  | **nAbs for challenge strain** | **IFN-γ** | **IL-2** | **Gzm B** |  | **nAbs for challenge strain** | **IFN-γ** | **IL-2** | **Gzm B** |  |
| **EIAV_HD** | **EIAV_HD_2** |  | N | 1.9x103 | N* |  | 54 | 2.45 | 3.55 | 1.234823 |  | 56 | 6.785 | 4.81 | 1.527423 |  |
|  | **EIAV_HD_8** |  | N | 1.54x103 | N* |  | 55 | 3.74 | 3.53 | 1.180692 |  | 31 | 5.415 | 3.73 | 1.534738 |  |
|  | **EIAV_HD_14** |  | N | 9.28x102 | N* |  | 23 | 2.635 | 3.46 | 1.177766 |  | 83 | 7.94 | 4.025 | 1.567655 |  |
|  | **EIAV_HD_5** |  | N | 5.5x103 | N* |  | NA | 2.545 | 7.11 | 1.212147 |  | 57 | 5.83 | 4.19 | 1.798078 |  |
|  | **EIAV_HD_3** |  | Y | 5.45x105 | N* |  | NA | 2.65 | 3.05 | 1.217267 |  | 35 | 5.97 | 4.735 | 1.558146 |  |
| **EIAV_MD** | **EIAV_MD_6** |  | N | 1.02x10^3^ | N* |  | 9 | 2.075 | 4.47 | 1.199711 |  | 46 | 4.31 | 6.36 | 1.625444 |  |
|  | **EIAV_MD_1** |  | N | 6.55x10^2^ | N* |  | 11 | 2.03 | 4.525 | 1.212878 |  | 26 | 5.225 | 4.88 | 1.648121 |  |
|  | **EIAV_MD_4** |  | N | 2.3x10^4^ | N* |  | NA | 2.15 | 4.915 | 1.141922 |  | 43 | 4.595 | 5.415 | 1.634953 |  |
|  | **EIAV_MD_13** |  | Y | 1.5x10^7^ | Y* |  | 22 | 1.955 | 4.06 | 1.215804 |  | 48 | 5.245 | 4.63 | 1.539858 |  |
|  | **EIAV_MD_16** |  | Y | 1.2x10^6^ | Y* |  | NA | 1.61 | 5.535 | 1.151432 |  | 41 | 5.675 | 7.46 | 1.602036 |  |
| **EIAV_LD** | **EIAV_LD_11** |  | Y | 5.63x10^6^ | N* |  | NA | 2.2 | 4.34 | 1.155089 |  | 39 | 2.23 | 4.335 | 1.553757 |  |
|  | **EIAV_LD_7** |  | Y | 2.34x10^6^ | N* |  | NA | 2.165 | 4.1 | 1.373808 |  | 30 | 2.38 | 3.78 | 1.596184 |  |
|  | **EIAV_LD_10** |  | N | 5.77X10^4^ | N* |  | NA | 1.8 | 4.08 | 1.161673 |  | 32 | 2.095 | 3.945 | 1.528154 |  |
|  | **EIAV_LD_9** |  | Y | 7.62x10^6^ | Y* |  | NA | 2.045 | 3.555 | 1.275056 |  | NA | 2.04 | 5.85 | 1.612277 |  |
|  | **EIAV_LD_21** |  | Y | 8.12x10^6^ | Y* |  | NA | 2.35 | 3.745 | 1.299195 |  | NA | 2.09 | 3.905 | 1.801736 |  |

**Table S1. Characterization of immune response induced by *env* diversity-varied strains before and after challenge.**

N indicated rectal temperature above 39°C. Y indicated rectal temperature under 39°C.N* indicated survivors of whom were challenged were euthanized at the end of the experiment. Y* indicated animals were dead induced by EIAV virulence challenged. nAbs: Neutralizing antibodies; Thee numbers of nAbs indicated the antibody in serum inhibition activity for EIAV challenge strain (EIAV_LN40_) at 10X dilution ; Positive neutralization determined as reciprocal titers>4. NA indicates not available. IFN-γ, IL-2 and GzmB reflect the cellular immune response.

**Table S2. The correlation between *env* diversity (SNP) and nAbs against challenge strain and immune cytokines.**

| **Stage** | **Item** | **SNP (diversity)** | **nAbs** | **IFN-γ** | **IL-2** | **GzmB** |
| --- | --- | --- | --- | --- | --- | --- |
| **6+ month before challenge** | **SNP (diversity)** |  |  |  |  |  |
|  | **nAbs** | **0.5932**** |  |  |  |  |
|  | **IFN-γ** | **0.6441**** | **0.625** |  |  |  |
|  | **IL-2** | **0.0327** | **-0.3613** | **-0.2723** |  |  |
|  | **GzmB** | **-0.2795** | **-0.0972** | **-0.0005** | **-0.236** |  |
| **0.5+ month after challenge** | **SNP (diversity)** |  |  |  |  |  |
|  | **nAbs** | **0.633**** |  |  |  |  |
|  | **IFN-γ** | **0.8973***** | **0.759** |  |  |  |
|  | **IL-2** | **-0.1089** | **-0.0345** | **0.109*** |  |  |
|  | **GzmB** | **-0.1035** | **-0.2709** | **-0.1609** | **-0.0188** |  |

Note: ****p*<0.001, ***p*<0.01, **p*<0.05
